# Biofunctionalized Nanostructured Zirconia for Biomedical Application: A Smart Approach for Oral Cancer Detection

**DOI:** 10.1002/advs.201500048

**Published:** 2015-06-03

**Authors:** Suveen Kumar, Saurabh Kumar, Sachchidanand Tiwari, Saurabh Srivastava, Manish Srivastava, Birendra Kumar Yadav, Saroj Kumar, Thien Toan Tran, Ajay Kumar Dewan, Ashok Mulchandani, Jai Gopal Sharma, Sagar Maji, Bansi Dhar Malhotra

**Affiliations:** ^1^ Nanobioelectronics LaboratoryDepartment of BiotechnologyDelhi Technological UniversityNew Delhi110042India; ^2^ Department of Physics and AstrophysicsUniversity of DelhiNew Delhi110007India; ^3^ Rajiv Gandhi Cancer Institute and Research CentreRohiniNew Delhi110085India; ^4^ Department of Chemical and Environmental EngineeringUniversity of CaliforniaRiversideCA92521USA

**Keywords:** biosensors, CYFRA‐21‐1, noninvasive detection, oral cancer, zirconia

## Abstract

Results of the studies are reported relating to application of the silanized nanostructured zirconia, electrophoretically deposited onto indium tin oxide (ITO) coated glass for covalent immobilization of the monoclonal antibodies (anti‐CYFRA‐21‐1). This biosensing platform has been utilized for a simple, efficient, noninvasive, and label‐free detection of oral cancer via cyclic voltammetry technique. The results of electrochemical response studies conducted on bovine serum albumin (BSA)/anti‐CYFRA‐21‐1/3‐aminopropyl triethoxy silane (APTES)/ZrO_2_/ITO immunoelectrode reveal that this immunoelectrode can be used to measure CYFRA‐21‐1 (oral cancer biomarker) concentration in saliva samples, with a high sensitivity of 2.2 mA mL ng^−1^, a linear detection range of 2–16 ng mL^−1^, and stability of six weeks. The results of these studies have been validated via enzyme‐linked immunosorbent assay.

## 1 Introduction

Oral cancer (OC) is presently one of the most prevalent cancers known till date, and it occurs more often in men than women. OC occurs as a sore in the mouth that does not easily heal. This cancer can be life threatening if not detected and treated early. It includes cancers of the floor of the mouth, tongue, cheeks, lips, sinuses, throat, etc. Smoking, chewing tobacco, alcohol consumption, diet (red and processed meat, fried foods), gastro‐esophageal reflux disease, human papillomavirus, and exposure to certain chemicals (e.g. asbestos, sulfuric acid, and formaldehyde) are some of the common causes behind the onset of OC.^1^, ^2^ These high risk factors are known to alter the expression of p16, APC, and p53 genes and account for the origin of OC.^3^ Most patients do not show any symptoms at the early stage. However, with passage of time, symptoms like mouth ulcer, loosening of teeth, and hoarse voice are known to develop.^1^

Many techniques, including laser capture microdissection, visualization adjuncts, cytopathology, and biopsy, are being used for detection and monitoring of OC.^4^, ^5^, ^6^ These methods are invasive, time‐consuming, expensive, and labor‐intensive. In this context, biosensors offer a reliable, user‐friendly, increased assay speed, high sensitivity and require low sample volumes.^7^, ^8^

Among the various nanomaterials, the nanostructured metal oxides have recently aroused much interest since these interesting materials provide high surface area for effective immobilization of desired biomolecules with desired orientation.^9^ In this context, nanostructured zirconium oxide (ZrO_2_) has been found to have interesting characteristics for biosensing applications. These characteristics include biocompatibility, excellent electrical, and surface charge properties that can be beneficial for integration of the immobilized biomolecules. Furthermore, oxygen moieties in ZrO_2_ may facilitate covalent attachment of both silane compound and indium tin oxide coated glass (ITO) surface.^10^, ^11^, ^12^

The detection of cancer via biomarkers has been recently proposed.^13^ In this context, the biomarkers, such as interleukin‐8 (IL‐8), interleukin‐6 (IL‐6), vascular endothelial growth factor (VEGF) and epidermal growth factor receptor (EGFR) are currently being used for detection of oral cancer.^14^, ^15^, ^16^, ^17^ The OC detection via these biomarkers is currently a challenge since these are secreted at very low concentration (≈pg mL^−1^) and the biosensors proposed for the detection of these biomarkers require serum samples and are thus invasive.^14^, ^15^, ^16^, ^17^ Thus, detection of OC biomarkers in human saliva samples provides a noninvasive and pain‐free alternative.

CYFRA‐21‐1 is a water‐soluble proteinaceous biomarker^18^ that represents a fragment of 40 kD of cytokeratin 19.^19^ The cutoff concentration of CYFRA‐21‐1 in saliva for normal subjects is 3.8 ng mL^−1^, whereas oral cancer patients have been found to have CYFRA‐21‐1 concentration as high as 17.46 ± 1.46 ng mL^−1^.^18^, ^20^ The recent developments in clinical research have identified salivary CYFRA‐21‐1, a reliable biomarker for the detection of OC.^18^, ^20^ To the best of our knowledge, there is no report till date pertaining to a biosensor for detection of OC via salivary CYFRA‐21‐1. Compared to the lower levels (≈pg mL^−1^) of afore‐mentioned biomarkers, CYFRA‐21‐1 is present in high concentration (≈ng mL^−1^), in saliva. Additionally, a biosensor for detection of CYFRA‐21‐1 from saliva samples is an attractive alternative with the immense potential for developing the biosensor for in‐home testing.^21^

Efforts have been made for the detection of OC utilizing biosensor platforms.^14^, ^15^, ^16^, ^17^ Yang et al. reported electrochemical biosensor for the detection of trace level of salivary IL‐8 protein.^15^ To achieve low level of detection, streptavidin and HRP labeling of the receptor biomolecules has been proposed which increases the complexity of detection.^15^ In another approach, CaCO_3_ nanoparticles based biosensor has been reported for IL‐6 detection in serum samples.^17^ Apart from being invasive, this method is complicated since it requires tagging of the receptor biomolecule.

We report results of the studies relating to development of a simple, label‐free and noninvasive anti‐CYFRA‐21‐1‐based electrochemical biosensor based on ZrO_2_ as transducer surface for OC detection in saliva samples. The results obtained using this electrochemical biosensor have been validated by performing enzyme‐linked immunosorbent assay (ELISA).^18^, ^20^

## 2 Results and Discussion

### 2.1 Structural and Morphological Studies

**Figure 1:**
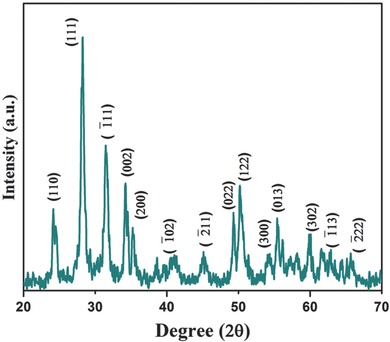
XRD pattern of the ZrO_2_ nanoparticles.

**Scheme 1:**
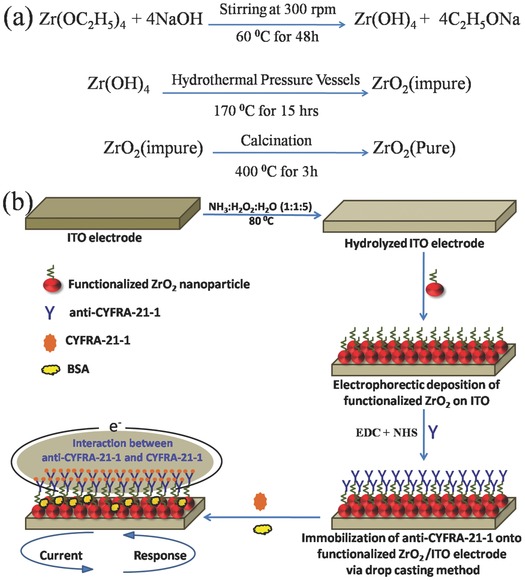
a) Synthesis mechanism of ZrO_2_ nanoparticles and b) schematic of the fabrication of BSA/anti‐CYFRA‐21‐1/APTES/ZrO_2_/ITO immunoelectrode for detection of oral cancer.

X‐ray diffraction (XRD) pattern of the synthesized product is shown in **Figure** **1**. The diffraction peaks corresponding to (110), (111), (1$\bar{1}$1), (022), (200), ($\bar{1}$02), ($\bar{2}$11), (022), (122), (300), (013), (302), ($\bar{1}$13), and ($\bar{2}$22) planes are well indexed with JCPDS NO. 37‐1484, indicating the formation of the monoclinic crystal phase of ZrO_2_ 22 and no peak corresponding to any impure phase is found in the XRD pattern. The XRD pattern of nanostructured zirconia samples prior to calcination is given in Figure S1 (Supporting Information), wherein an additional peak is observed at 30.37° due to (111) reflection of the sodium ethoxide (JCPDF No. 31‐1885), formed as an intermediate product (**Scheme** **1**a). However, after calcination, this peak disappears suggesting removal of the impurities. It can be seen (Figure S1, Supporting Information) that the (111) plane has the highest intensity revealing that the crystallites are grown more orderly along the (111) plane. The average crystallite size *“D”* has been estimated to be ≈50 nm using the Scherer formula (Equation (1))

(1) $$D=\frac{0.9\lambda }{\beta \cos\theta }$$

where *λ =* 1.54060 Å is the wavelength of the target Cu‐Kα, *θ* is the Bragg's diffraction angle, and *β* is the full width at half maximum (FWHM) of the diffraction peak.

**Figure 2:**
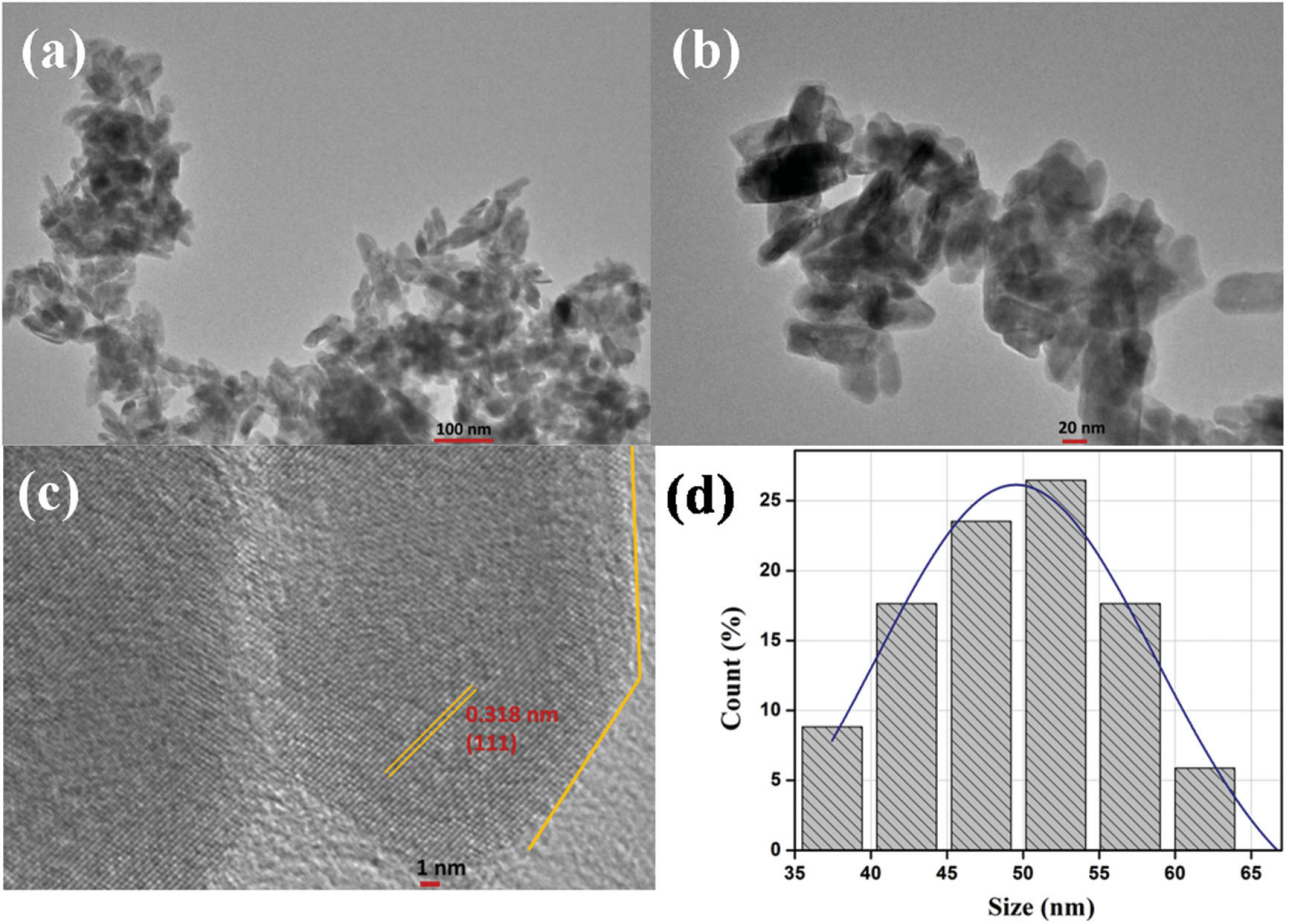
a,b) TEM image, c) HR‐TEM image, and d) particle size distributionof ZrO_2_ nanoparticles.

The size and shape of the ZrO_2_ nanoparticles have been investigated using transmission electron microscope (TEM) (**Figure** **2**a,b). The TEM image of the nanoparticles reveals that particles have hexagonal and sheet‐type structure. The individual ZrO_2_ nanoparticles have been selected for HR‐TEM analysis (Figure 2c). The HR‐TEM image suggests that these particles are polycrystalline in nature. The lattice fringes with a *d‐*spacing of 0.318 nm corresponding to (111) planes are clearly visible. These observations are in agreement with the *d*‐values calculated for the respective planes from the XRD studies. In order to analyze the size distribution quantitatively, the histogram is fitted with the Gaussian function and the mean size of the particle is calculated to be 50 nm (Figure 2d).

**Figure 3:**
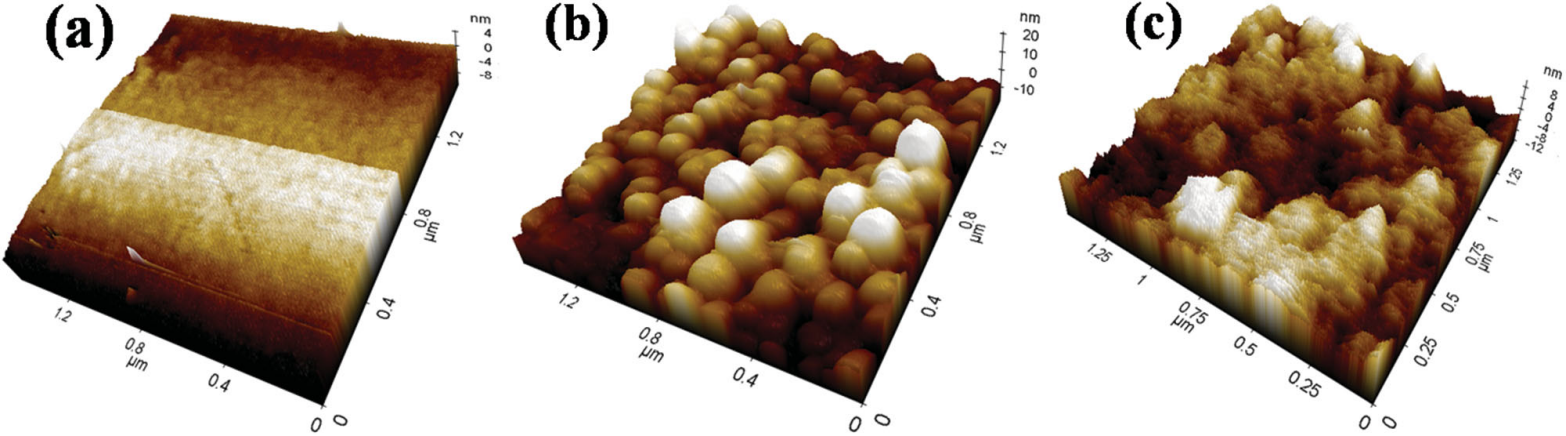
AFM image of a) APTES/ZrO_2_/ITO electrode, b) anti‐CYFRA‐21‐1/APTES/ZrO_2_/ITO bioelectrode, and c) BSA/anti‐CYFRA‐21‐1/APTES/ZrO_2_/ITO bioelectrode.

The results of surface morphology studies conducted on APTES/ZrO_2_/ITO, anti‐CYFRA‐21‐1/APTES/ZrO_2_/ITO, and BSA/anti‐CYFRA‐21‐1/APTES/ZrO_2_/ITO electrodes via atomic force microscopy (AFM) are shown in **Figure** **3**a–c. The AFM image of the APTES/ZrO_2_/ITO film (Figure 3a) shows smooth surface with an average roughness of ≈1.9 nm. This suggests the formation of a uniform layer due to intermolecular interaction between APTES/ZrO_2_ and ITO surfaces. Figure 3b shows AFM image of the anti‐CYFRA‐21‐1 immobilized on APTES/ZrO_2_/ITO electrode surface with average roughness of ≈16.9 nm. The increased roughness may be attributed to globular Y‐shaped structure of the antibodies and the immobilization of BSA onto the surface of anti‐CYFRA‐21‐1/APTES/ZrO_2_/ITO, resulting in decrease of the surface roughness to ≈2.7 nm (Figure 3c). The decreased roughness may be attributed to the nonspecific adsorption of BSA onto exposed APTES/ZrO_2_ sites. Figure S2 (Supporting Information) shows side view of the SEM image of electrophoretically deposited APTES/ZrO_2_/ITO film indicating presence of the uniformly dispersed grains of APTES functionalized ZrO_2_ nanoparticles at ITO substrate. However, in case of anti‐CYFRA‐21‐1/APTES/ZrO_2_/ITO bioelectrode the granular morphology becomes globular due to covalent attachment of antibodies to the APTES/ZrO_2_. Further, due to high anti‐CYFRA‐21‐1 loading, the individual grains of nanoparticles are not observed.

**2.2. Fourier Transformed Infrared Spectroscopic (FT‐IR) and X‐Ray Photoelectron Spectroscopy (XPS) Studies**

**Figure 4:**
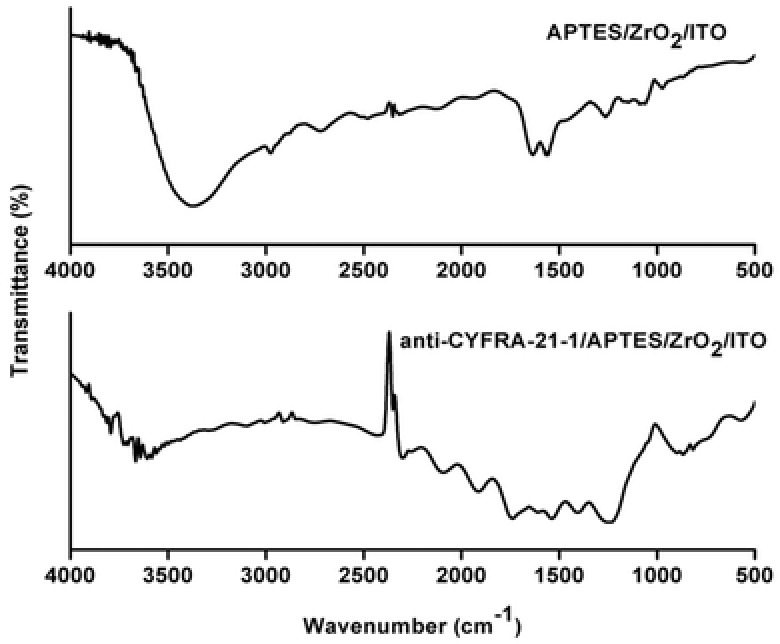
FT‐IR spectra of APTES/ZrO_2_/ITO and anti‐CYFRA‐21‐1/APTES/ZrO_2_/ITO electrode.

FT‐IR spectrum (**Figure** **4**) of APTES/ZrO_2_/ITO electrode exhibits characteristic peaks at 540 cm^−1^ due to symmetric stretching of the Zr—O bonds.^23^ The bands seen between 950 and 1250 cm^−1^ are due to Si—O—Zr and Si—O—C modes.^24^ The bands found at 1566 and 1629 cm^−1^ are due to scissor and stretching mode of NH_2_ present in the APTES.^24^ Furthermore, a broad band present at 3377 cm^−1^ can be ascribed to the N—H stretching vibration along with —OH stretching of water mole­cules. It may be noted that intensity of the 1566 and 1629 cm^−1^ bands (corresponding to ‐NH_2_ of APTES/ZrO_2_/ITO) is considerably reduced in case of anti‐CYFRA‐21‐1/APTES/ZrO_2_/ITO. These results reveal covalent attachment of antibodies with amino terminal of APTES/ZrO_2_/ITO. The band seen at 1400 cm^−1^ is due to bending vibration of the CH_2_ aliphatic moiety of antibodies. Further, bands at 1538 and 1740 cm^−1^ correspond to amide II and —C = O stretching (carboxylic group) of the antibody molecules.^24^, ^25^, ^26^

**Figure 5:**
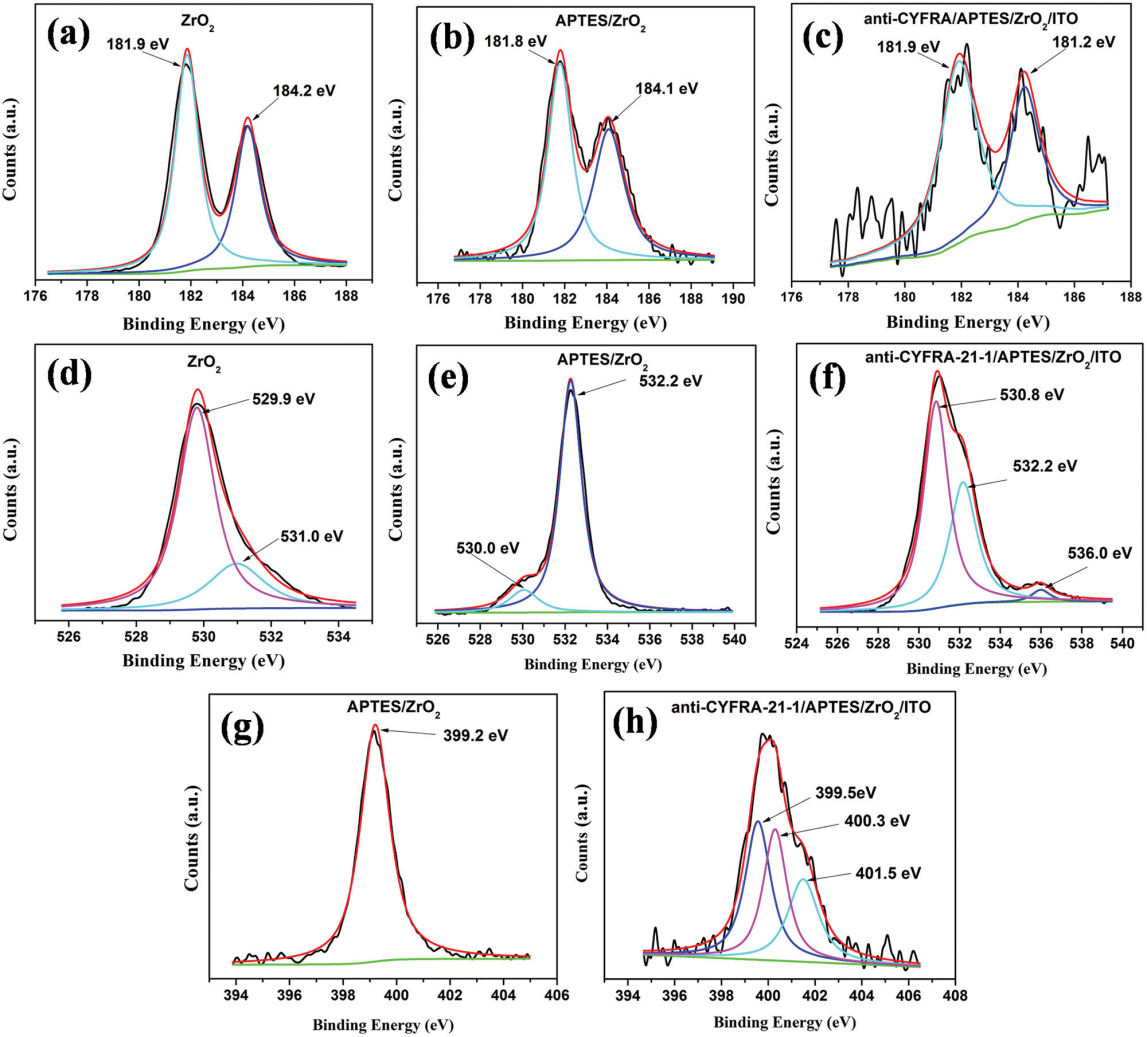
Zr 3d XPS spectra of a) ZrO_2_, b) APTES/ZrO_2_, c) anti‐CYFRA‐21‐1/APTES/ZrO_2_, O 1s spectra of d) ZrO_2_, e) APTES/ZrO_2_, f) anti‐CYFRA‐21‐1/APTES/ZrO_2_, N 1s spectra of g) APTES/ZrO_2_ and h) anti‐CYFRA‐21‐1/APTES/ZrO_2_.

Analysis of the XPS studies was performed by first calibrating the binding energy (BE) levels to the C 1s spectrum, using the sp^3^ carbon (C—C) peak from adventitious carbon at 284.8 eV. **Figure** **5**a–c shows XPS of Zr 3d spectrum of ZrO_2_, APTES/ZrO_2_ and anti‐CYFRA‐21‐1/APTES/ZrO_2_/ITO electrodes. The XPS spectra of all three samples have been deconvoluted into the corresponding binding energy peaks. The observed spin–orbit doublet peaks seen at around 181.9 and 184.2 eV correspond to Zr 3d_5/2_ and Zr 3d_3/2_ electrons, respectively, and are due to the presence of Zr in its oxidized state, Zr^4+^ from ZrO_2_.^27^ Figure 5d shows O 1s spectrum of ZrO_2_ containing a maximum around 530 eV suggesting the presence of electrons from oxygen in the O^2−^ state from ZrO_2_. Further, the peak seen at higher binding energy position of 531.0 eV may perhaps be due to other chemical states of oxygen (C‐O or C=O). Figure 5e shows the O 1s spectrum of APTES modified ZrO_2_ where two peaks at 530.0 and 532.2 eV are observed. The peak at higher binding energy (532.2 eV) is assigned to the oxygen atom linked to silicon, while the other one at lower binding energy (530.0 eV) is attributed to the oxygen in ZrO_2_.^28^ In case of the anti‐CYFRA‐21‐1/APTES/ZrO_2_/ITO immunoelectrode, the deconvoluted O 1s spectrum (Figure 5f) shows binding energy peaks around 530.8, 532.2, and 536.0 eV. The additional peak at 536.0 eV is attributed to the amide oxygen present in the antibody molecules. Figure 5g shows nitrogen core level XPS of APTES modified ZrO_2_ where the BE peak seen at 399.2 eV is attributed to the nitrogen present in the APTES. In case of the anti‐CYFRA‐21‐1/APTES/ZrO_2_/ITO, three distinct peaks are observed at 399.5, 400.3, and 401.5 eV (Figure 5h). The peak observed at the binding energy of 401.5 eV is attributed to the ≡N species, whereas another peak seen at lower binding energy of 400.3 eV corresponds to amide nitrogen (OC—NH) present in the antibody molecules.^29^

#### 2.3 Electrochemical Studies

**Figure 6:**
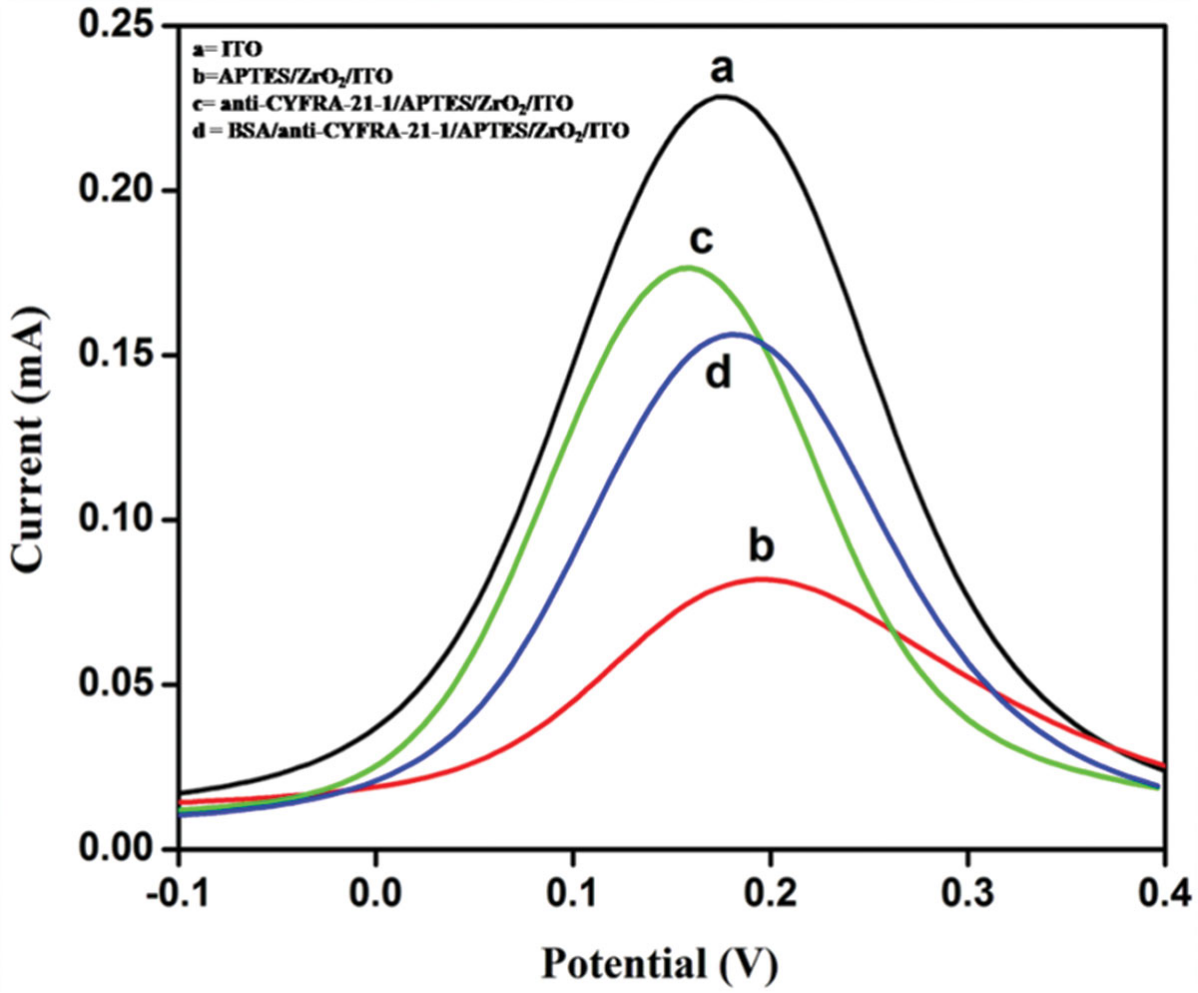
Differential pulse voltammogram (DPV) of a) ITO, b) APTES/ZrO_2_/ITO, c) anti‐CYFRA‐21‐1/APTES/ZrO_2_/ITO, and d) BSA/anti‐CYFRA‐21‐1/APTES/ZrO_2_/ITO electrodes.

**Figure** **6** shows results of differential pulse voltammetry (DPV) studies conducted on ITO, APTES/ZrO_2_/ITO, anti‐CYFRA‐21‐1/APTES/ZrO_2_/ITO, and BSA/anti‐CYFRA‐21‐1/APTES/ZrO_2_/ITO electrodes, respectively. It can be seen that magnitude of current obtained for APTES/ZrO_2_/ITO electrode (0.082 mA) is lower than that of bare ITO electrode (0.229 mA), indicating decreased electron transfer between solution and APTES/ZrO_2_/ITO interface. However, after the immobilization of anti‐CYFRA‐21‐1 onto the APTES/ZrO_2_/ITO electrode, the magnitude of peak current (0.176 mA) increases and the peak potential shifts to a lower value indicating facile electron transfer to the electrode surface. The magnitude of current response increases after anti‐CYFRA‐21‐1 functionalizes the APTES/ZrO_2_/ITO electrode surface. The APTES/ZrO_2_ perhaps acts as a mediator on the electrode surface and it can significantly shorten the electron tunneling distance between the antibodies and the electrode resulting in a higher current. Besides this, the electrostatic interaction between the free site of the antibodies (‐NH_2_ terminal) and the redox species may result in fast electron diffusion toward the immunoelectrode.^30^ After BSA immobilization, the magnitude of current decreases to 0.156 mA. This is due to nonspecific adsorption of BSA onto the exposed APTES/ZrO_2_ sites of the anti‐CYFRA‐21‐1/APTES/ZrO_2_/ITO immunoelectrode. After the BSA treatment, the peak potential shifts toward the higher value due to insulating characteristics of the BSA molecules.

The effect of pH on the electrochemical response of the BSA/anti‐CYFRA‐21‐1/APTES/ZrO_2_/ITO electrode has been investigated using cyclic voltammetry (CV) at scan rate of 20 mV s^−1^ in phosphate buffer saline (PBS) containing 5 × 10^−3^ m [Fe(CN)_6_]^3−/4−^. It can be seen that magnitude of the current response is maximum at neutral pH (7.0) (Figure S3, Supporting Information). This may perhaps be due to the fact that biological molecules such as amino acid, enzyme, antigen, antibody, etc. are present in natural form with high activity at neutral pH. However, in acidic or basic medium antibodies get denatured due to the interaction of H^+^ or OH^−^ ion on amino acid sequence of antibodies.^31^, ^32^ Consequently, all electrochemical studies have been conducted using PBS of pH 7.0 with 5 × 10^−3^ m [Fe(CN)_6_]^3−/4−^. Figures S4 and S5 (Supporting Information) show CV response of APTES/ZrO_2_/ITO and BSA/anti‐CYFRA‐21‐1/APTES/ZrO_2_/ITO electrode as a function of scan rate (10–100 mV s^−1^), respectively. We observe that both cathodic (*I*_pc_) and anodic (*I*_pa_) peak current vary linearly with square root of the scan rate (insets a) in Figures S4 and S5 in the Supporting Information), indicating that electrochemical reaction is a diffusion‐controlled process.^30^ The slopes and intercepts are given by Equations (2)–(5)

(2) $$x00026;x00026;I_{{\mathrm{pc}(\mathrm{APTES}/\mathrm{ZrO}}_{2}/\mathrm{ITO})}=[9.32\;\mu A(s\;{\mathrm{mV}}^{‐1})\;x00026;x00026;\;\;\times (\mathrm{scan}\;\mathrm{rate}\;[\mathrm{mV}\;s^{‐1}{])}^{1/2}]+7.96\;\;\mu A,R^{2}=0.998$$

(3) $$x00026;x00026;I_{{\mathrm{pa}(\mathrm{APTES}/\mathrm{ZrO}}_{2}/\mathrm{ITO})}=‐[8.43\;\mu A(s\;{\mathrm{mV}}^{‐1})\;x00026;x00026;\;\;\times (\mathrm{scan}\;\mathrm{rate}\;[\mathrm{mV}\;s^{‐1}{])}^{1/2}]‐8.29\;\;\mu A,R^{2}=0.999$$

(4) $$x00026;x00026;I_{{\mathrm{pc}(\mathrm{BSA}x0002F;anti‐CYFRA‐21‐1/APTES/ZrO}_{2}/\mathrm{ITO})}=[52.54\;\mu A(s\;{\mathrm{mV}}^{‐1})\;x00026;x00026;\;\;\times (\mathrm{scan}\;\mathrm{rate}\;[\mathrm{mV}\;s^{‐1}{])}^{1/2}]+19.60\;\mu A,R^{2}=0.992$$

(5) $$x00026;x00026;I_{{\mathrm{pa}(\mathrm{BSA}x0002F;anti‐CYFRA‐21‐1/APTES/ZrO}_{2}/\mathrm{ITO})}=‐[44.50\;\mu A(s\;{\mathrm{mV}}^{‐1})\;x00026;x00026;\;\;\times (\mathrm{scan}\;\mathrm{rate}\;[\mathrm{mV}\;s^{‐1}{])}^{1/2}]‐80.60\;\mu A,R^{2}=0.996$$

The difference of cathodic (*E*_pc_) and anodic (*E*_pa_) peak potentials (Δ*E*_p_ =*E*_pc_ −*E*_pa_) and square root of scan rate for APTES/ZrO_2_/ITO and BSA/anti‐CYFRA‐21‐1/APTES/ZrO_2_/ITO electrodes exhibit a linear relationship and are given by Equations (6) and (7). A good linear fitting suggests a facile electron transfer from medium to electrode (inset b in Figures S4 and S5 in the Supporting Information)

(6) $$x00026;x00026;\Delta E_{p}(V{)}_{{\mathrm{APTES}/\mathrm{ZrO}}_{2}/\mathrm{ITO}}=[0.035\;V(s\;{\mathrm{mV}}^{‐1})\;x00026;x00026;\;\;\times (\mathrm{scan}\;\mathrm{rate}\;[\mathrm{mV}\;s^{‐1}{])}^{1/2}]+0.10\;V,R^{2}=0.995$$

(7) $$x00026;x00026;\Delta E_{p}(V{)}_{{\mathrm{BSA}x0002F;anti‐CYFRA‐21‐1/APTES/ZrO}_{2}/\mathrm{ITO}}=[0.035\;V(s\;{\mathrm{mV}}^{‐1})\;x00026;x00026;\;\;\times (\mathrm{scan}\;\mathrm{rate}\;[\mathrm{mV}\;s^{‐1}{])}^{1/2}]+0.27\;V,R^{2}=0.992$$

Figure S6 (Supporting Information) shows the variation of current obtained via CV on interaction of CYFRA‐21‐1 with the BSA/anti‐CYFRA‐21‐1/APTES/ZrO_2_/ITO immunoelectrode for different incubation times. We observe an increase in current with respect to incubation time from 2 to 20 min. However, after 20 min of sample incubation the current becomes nearly constant. Thus, we have selected the sample incubation time as 20 min during which the antigen–antibody binding reaction reaches the steady state.

#### 2.4 Response Studies

**Figure 7:**
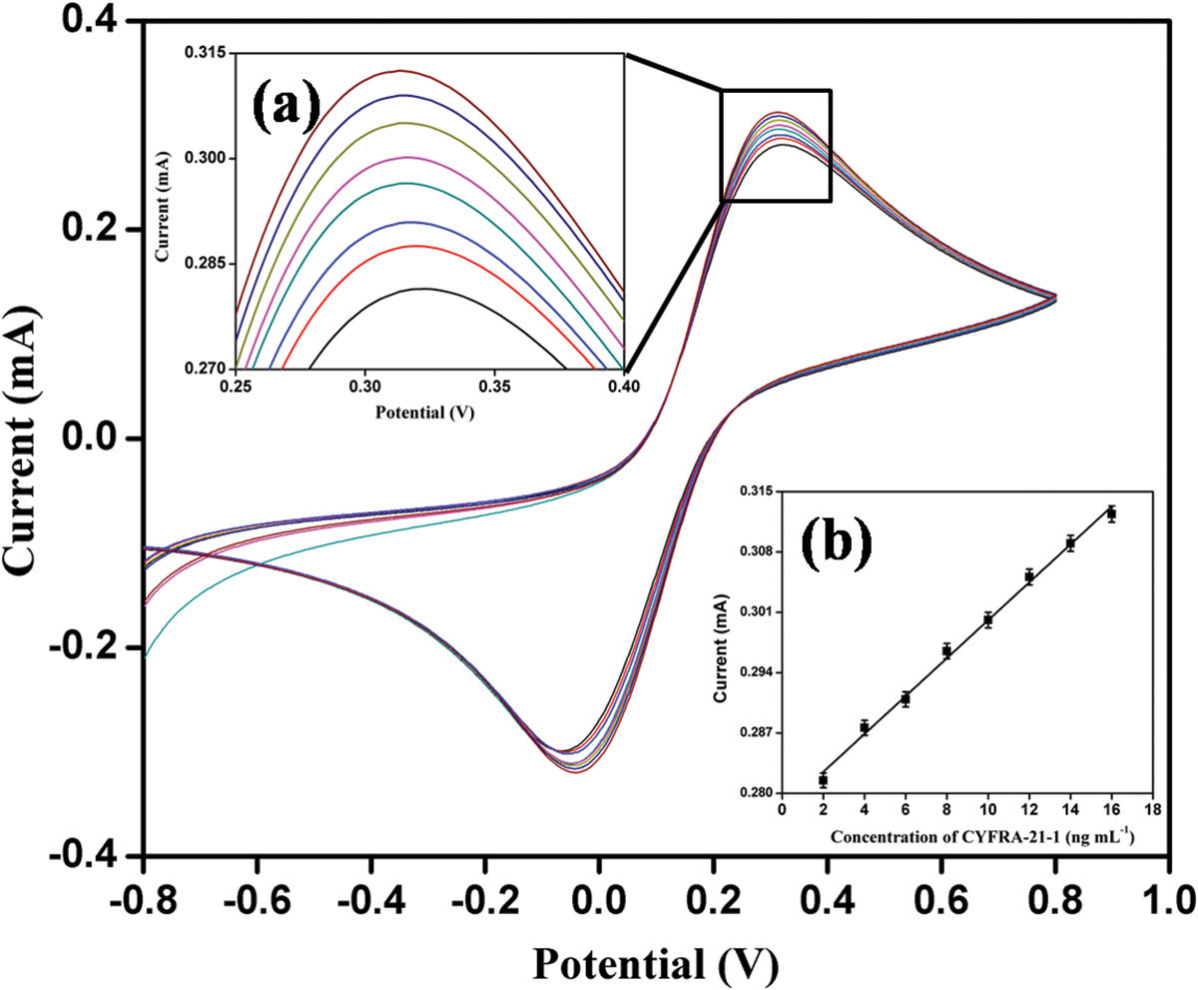
Electrochemical response of BSA/anti‐CYFRA‐21‐1/APTES/ZrO_2_/ITO immunoelectrode as a function of CYFRA‐21‐1 concentration (2–16 ng mL^−1^). The magnified view of oxidation peak current (inset a)), calibration curve between magnitude of peak current and concentration of CYFRA‐21‐1 (ng mL^−1^) (inset b)).

The electrochemical response of BSA/anti‐CYFRA‐21‐1/APTES/ZrO_2_/ITO immunoelectrode has been measured as a function of CYFRA‐21‐1 concentration (2–16 ng mL^−1^) in PBS (50 × 10^−3^ m, pH 7.0, 0.9%NaCl) containing [Fe(CN)_6_]^3−/4−^ (5 × 10^−3^ m) at 20 mV s^−1^ (scan rate) using the CV technique (**Figure** **7**). All the measurements have been repeated three times for each concentration. It can be seen that magnitude of the oxidation current increases with increase in concentration of CYFRA‐21‐1. This electrochemical response behavior can be assigned to the formation of an antigen–antibody complex between CYFRA‐21‐1 and anti‐CYFRA‐21‐1 antibody on the surface of the immunoelectrode resulting in the formation of an electron transfer accelerating layer.^33^ Under optimized assay conditions, the calibration curve using CYFRA‐21‐1 standard shows a linear response from the BSA/anti‐CYFRA‐21‐1/APTES/ZrO_2_/ITO immunoelectrode in the concentration range of 2–16 ng mL^−1^ (Figure 7, inset b) with a linear regression coefficient of 0.995 as given in following equation

(8) $$\begin{array}{l} I_{p}=[2.2\;\mathrm{mA}\;\mathrm{mL}\;{\mathrm{ng}}^{-1})\times (\mathrm{conc}.\;\mathrm{of}\;\mathrm{CYFRA‐21‐1}({\mathrm{ngmL}}^{-1}))]\\ \;\;\;\;+0.\mathrm{278}\;\mathrm{mA},R^{2}=0.\mathrm{995} \end{array}$$

The sensitivity of the fabricated BSA/anti‐CYFRA‐21‐1/APTES/ZrO_2_/ITO immunoelectrode has been estimated to be 2.2 mA mL ng^−1^ and the lower detection limit (LD) has been determined to be 0.08 ng mA^−1^ mL^−1^ using Equation (9)

(9) $$\mathrm{Detection}\;\mathrm{limit}=3\sigma_{b}/m$$

where *σ*_b_ is standard deviation and *m* is the slope of the curve.

A control experiment has been conducted using the APTES/ZrO_2_/ITO electrode as a function of CYFRA‐21‐1 concentration (Figure S7, Supporting Information). We do not observe any significant change in the current response of the APTES/ZrO_2_/ITO electrode with increasing concentration of CYFRA‐21‐1 antigen. It appears that the APTES/ZrO_2_/ITO electrode surface does not interact with antigen molecule and therefore the electrochemical current remains unchanged. Next, we have performed the interferent studies conducted on BSA/anti‐CYFRA‐21‐1/APTES/ZrO_2_/ITO immumoelectrode using various potentially interfering substances (Figure S8, Supporting Information). Human saliva contains various analytes, such as carcinoembroyonic antigen (CEA) [4–16 ng mL^−1^], cardiac troponin I (Tn‐I) [0.19 ng mL^−1^], sodium carboxymethyl cellulose (NaCMC) [10 mg mL^−1^], glucose [7 mg mL^−1^], etc. We find that magnitude of the oxidation current decreases by 0.14%, 1.3%, 1.68%, and 1.75% upon addition of CEA, NaCMC, Tn‐I, and glucose, respectively, indicating high selectivity of the biosensor.

The shelf‐life of BSA/anti‐CYFRA‐21‐1/APTES/ZrO_2_/ITO immunoelectrode (Figure S9, Supporting Information) has been determined by measuring the CV response in standard solution of CYFRA‐21‐1 (6 ng mL^−1^) in PBS at one week interval. It is found that no significant change occurs in the peak current up to about six weeks after which decrease in the response current is observed.

The effect of addition of different amount of saliva sample on the response current was investigated and shown in Figure S10 (Supporting Information). Here, we have taken different quantities of artificial saliva^34^ (prepared as reported elsewhere) and observed the effect on electrochemical response of BSA/anti‐CYFRA‐21‐1/APTES/ZrO_2_/ITO immunoelectrode. It has been observed that electrochemical response was stable up to addition of 1000 μL artificial saliva and thereafter it tends to decrease toward higher concentration.

#### 2.5 Real Sample Analysis

Quantification of CYFRA‐21‐1 in saliva sample has been accomplished by ELISA in triplicate. The double‐antibody sandwich ELISA is performed in microtiter wells precoated with anti‐CYFRA‐21‐1. After following all the steps, colorimetric reaction occurs and absorbance is recorded at 450 nm in ELISA plate reader. A series of CYFRA‐21‐1 concentration in saliva samples obtained by ELISA (**Table** **1**) have been used to test the accuracy of the fabricated biosensor. It can be seen that a reasonable correlation exists between magnitude of the CV current response of the fabricated immunoelectrode in the presence of (a) CYFRA‐21‐1 concentrations in saliva samples determined by ELISA and (b) standard concentration of CYFRA‐21‐1 (Table 1 and Figure S11, Supporting Information). The observed results exhibit acceptable %RSD (relative standard deviation) indicating high accuracy of the fabricated biosensor. The sensing characteristics of proposed immunosensor have been summarized in **Table** **2** along with those reported in the literature.

**Table 1:** Determination of CYFRA‐21‐1 concentration in saliva samples using BSA/anti‐CYFRA‐21‐1/APTES/ZrO_2_/ITO bioelectrode

S. No.	CYFRA‐21‐1 concentration determined using ELISA [ng mL^−1^]	Peak current [mA] obtained for standard CYFRA‐21‐1 samples	Peak current samples [mA] obtained with saliva	% RSD
1	15.35	0.312	0.314	0.45%
2	12.50	0.305	0.292	3.08%
3	12.70	0.306	0.295	2.59%
4	13.50	0.308	0.297	2.57%
5	14.15	0.310	0.299	2.55%
6	15.55	0.312	0.294	4.20%

**Table 2:** Comparative analysis of existing techniques with fabricated biosensor for oral cancer detection (LDR = linear detection limit, SL = shelf life, S = Sensitivity)

Method	Detection technique	Invasive/noninvasive	Label	Sample	Biomarker	Concentration range of biomarker	Detection limit/detection range	Response time	Ref.
Cytopathology	Staining	Invasive	–	Cells	–	–	–	1 week	5
Biopsy	Cell culture	Invasive	–	Tissue	–	–	–	2–3 weeks	4
Visualization adjuncts	Staining	Invasive	–	Tissue	–	–	–	1 week	4
	Surface plasmon resonance	Noninvasive	Yes	Saliva	IL‐8 (protein)	29.8–85.9 × 10^−12^ m	S:– LDR: 1–195 × 10^−12^ m SL:–	13 min	15
	Amperometric	Invasive	Yes	Serum	IL‐6 (protein)	≤6 pg mL^−1^ to ≥20 pg mL^−1^	S: 19.3 nA mL (pg IL‐6)^−1^ cm^−2^ LDR: 0.5–30 pg mL^−1^ SL:–	–	16
Biosensor	Differential pulse voltametry	Invasive	Yes	Serum	IL‐6 (protein)	<6 pg mL^−1^ to >20 pg mL^−1^	S:– LDR: 0.002–20 ng mL^−1^ SL: 1 month	–	17
	Chrono‐amperometry	Noninvasive	Yes	Saliva	has‐miR‐200a (mi‐RNA)	–	S:– LDR: 1 am to 10 f m SL:–	–	14
	Cyclic voltametry	Noninvasive	No	Saliva	CYFRA‐21‐1 (protein)	0–18 ng mL^−1^	S: 2.2 mA mL ng^−1^ LDR: 2–16 ng mL^−1^ SL: 6 weeks	20 min	Present work

## 3 Conclusion

We have fabricated a simple, efficient, label‐free, and noninvasive nanostructured zirconia based biosensing platform for oral cancer detection. ZrO_2_ nanoparticles have been synthesized via the hydrothermal method and its silanization has been achieved using APTES. Thin films of APTES/ZrO_2_/ITO have been fabricated via electrophoretic deposition and followed by covalent immobilization of antibodies. The salivary CYFRA‐21‐1 antigen has been used as a biomarker for the detection of oral cancer. In comparison with other reported oral cancer detection methods including biosensors, the BSA/anti‐CYFRA‐21‐1/APTES/ZrO_2_/ITO biosensor is simple, exhibits a wider detection range of 2–16 ng mL^−1^ high sensitivity of 2.2 mA mL ng^−1^, detection limit of 0.08 ng mL^−1^, and shelf‐life of six weeks. This novel saliva based oral cancer biomarker (CYFRA‐21‐1) opens a new window for noninvasive research in oral and other cancers.

## 4 Experimental Section

*Reagents*: Zirconium ethoxide [Zr(C_2_H_5_OH)_4_] and 1‐(3‐(dimethylamino)‐propyl)‐3‐ethylcarbodiimide hydrochloride (EDC) [C_8_H_17_N_3_] of AR grade were purchased from Sigma‐Aldrich. Sodium hydroxide (NaOH) pellets, cetyltrimethylammonium bromide (CTAB) [C_19_H_42_BrN], sodium monophosphate [NaH_2_PO_4_], sodium diphosphatedihydrate [Na_2_HPO_4_·2H_2_O], *N*‐hydroxysuccinimide (NHS) [C_4_H_5_NO_3_], sodium chloride [NaCl], potassium ferricyanide {K_3_[Fe(CN)_6_]}, and potassium ferrocyanide {K_4_[Fe(CN)_6_]3H_2_O} were procured from Fisher Scientific. The 3‐aminopropyl triethoxy silane (APTES) was purchased from Alfa‐aesar. All chemicals were of analytical grade and were used without any further purification. Phosphate buffered saline (PBS) solution of pH 7.0 was prepared using Na_2_HPO_4_·2H_2_O (0.05 mol L^−1^) and NaH_2_PO_4_ (0.05 mol L^−1^). Fresh PBS solution was prepared using Milli‐Q water having resistivity of 18 MΩ cm and stored at 4 °C. The CYFRA‐21‐1 and anti‐CYFRA‐21‐1 were obtained from Ray Biotech, Inc., India. These biomolecules were further diluted by using PBS buffer of pH 7.0. CYFRA‐21‐1 ELISA Kit was purchased from KinesisDX, USA. ELISA plate reader was from iMark, Bio red, USA.

*Apparatus*: The crystallinity and phase formation of the synthesized product were investigated via X‐ray diffraction (XRD) studies [Bruker D‐8 Advance]. A monochromatic X‐ray beam with Cu Kα radiation (*λ* = 1.5406 Å) was used to record the spectrum. Particle size and shape of ZrO_2_ nanoparticles were determined using transmission electron microscopy (TEM) on a JEOL JEM‐2100F‐TEM system. Surface morphology of APTES/ZrO_2_/ITO, anti‐CYFRA‐21‐1/APTES/ZrO_2_/ITO, and BSA/anti‐CYFRA‐21‐1/APTES/ZrO_2_/ITO electrodes was examined using atomic force microscopy (AFM) on a ParkXe‐100 AFM system. The functional groups and bonds present in APTES/ZrO_2_/ITO and anti‐CYFRA‐21‐1/APTES/ZrO_2_/ITO were investigated through Fourier transform infrared (FT‐IR) spectroscopy (Perkin‐Elmer, Model spectrum ATR accessory) and X‐ray photoelectron spectroscopy (XPS). XPS was carried out using a Kratos AXIS ULTRADLD and Kratos Axis‐Nova XPS system. The cyclic voltammetry (CV) and differential pulse voltammetry (DPV) studies were performed on an Autolab Potentiostat (Netherlands). These measurements were conducted using a three‐electrode system with ITO coated glass electrode as the working electrode, platinum (Pt) as the counter electrode, and silver‐silver chloride (Ag/AgCl) as the reference electrode. A PBS solution (50 × 10^−3^ m) of pH 7.0 containing 5 × 10^−3^ m of [Fe(CN)_6_^3−/4−^] as redox species was used as the electrolyte.

*Collection of Saliva Samples*: The saliva samples of oral cancer patient were obtained from Rajiv Gandhi Cancer Institute and Research Centre, Delhi (India). All saliva samples were collected under a protocol approved by Rajiv Gandhi Cancer Institute and Research Center Institutional Review Board (R. No. RGCIRC/IRB/60/2014) and all patients provided written consent. Ethical approval of the Institutional Ethical and Biosafety committee, DTU (R. No. BT/IEBC/2014/714) was also obtained. Samples were collected, processed, and stored under similar conditions. The unstimulated whole saliva was collected from six patients diagnosed for oral cancer. Table S1 (Supporting Information) indicates pathological data of oral cancer patients. Deionized water (5 mL) was used for rinsing of mouth and expectorated into sterilized tube, kept in ice condition. The collected saliva was centrifuged at 2800 rcf at room temperature for 30 min after which the supernatant was collected in a sterilized tube and stored at −20 °C until further used.

*Preparation and Functionalization of Nanostructured Monoclinic‐Zirconia (ZrO_2_) with APTES*: Nanostructrured ZrO_2_ was synthesized by the hydrothermal method. In brief, synthesis process involved the following steps: 0.2 m zirconium ethoxide, 0.8 m sodium hydroxide, and 0.1 m solution of CTAB were prepared in deionized water, separately. The prepared solution of CTAB was dropped into the solution of zirconium ethoxide with a constant stirring (300 rpm) at 60 °C for 2 h after which solution of sodium hydroxide was added and stirred for the next 2 h. The prepared solution was transferred into a Teflon vessel and placed in a stainless‐steel tank. This assembly was kept in an oven at 170 °C for 15 h for hydrothermal reaction. After completion of the reaction, hydrothermal reactor was allowed to cool to room temperature (25 °C). The precipitate obtained from the reaction was washed with deionized water through the centrifugation process until pH of the solution became neutral. Subsequently, thus‐obtained slurry was dried at 100 °C overnight and annealed at 400 °C for 3 h. This product was crushed into fine powder using the mortar‐pestle for further characterization. The schematic of the preparation of ZrO_2_ nanoparticles is shown in Scheme 1a.

*Functionalization of ZrO_2_*: 100 mg of ZrO_2_ was added to 20 mL of isopropanol and sonicated to obtain a highly dispersed suspension. Next, 200 μL of 98% APTES was mixed and stirred at 300 rpm for 48 h at room temperature (25 °C). In order to remove the unbound APTES, the suspension was filtered using Whatman paper and washed thoroughly with deionized water.

*Electrophoretic Deposition of Functionalized ZrO_2_ on ITO Electrode (APTES/ZrO_2_/ITO)*: APTES functionalized ZrO_2_ was deposited onto the prehydrolyzed ITO electrode via an electrophoretic deposition (EPD) technique using a Genetix, GX300C instrument. Prior to deposition, a colloidal suspension containing 10 mg of APTES/ZrO_2_ was prepared in 2 mL of C_2_H_5_OH and 48 mL of deionized water followed by ultrasonication. The ITO and platinum electrodes were kept 1 cm apart in this colloidal suspension. Next, a DC potential of 11 V was applied for 20 s for EPD of APTES/ZrO_2_ onto the ITO electrode. An optimized surface area of the APTES/ZrO_2_/ITO electrodes was determined to be 0.25 cm^2^. The as‐prepared electrode was washed with deionized water and dried at room temperature (25 °C) overnight.

*Immobilization of anti‐CYFRA‐21‐1 over APTES/ZrO_2_/ITO Electrode*: The anti‐CYFRA‐21‐1 solution (50 μg mL^−1^) was prepared in PBS (pH = 7.0). 15 μL of anti‐CYFRA‐21‐1 was mixed with 7.5 μL of 0.4 m EDC (activator) and 7.5 μL of 0.1 m NHS (coupling agent). Subsequently, this solution (30 μL) was uniformly spread onto APTES/ZrO_2_/ITO electrode by drop‐casting. The electrode was kept in a humid chamber at room temperature (25 °C) followed by washing with PBS to remove any unbound antibody molecules. The —COOH group of anti‐CYFRA‐21‐1 can be covalently bound with ‐NH_2_ terminal of APTES via strong amide bond (OC‐NH). Lastly, bovine serum albumin (BSA = 2 mg dL^−1^) was used for blocking the nonspecific active sites of the electrode. The BSA/anti‐CYFRA‐21‐1/APTES/ZrO_2_/ITO immunoelectrode was stored at 4 °C when not in use. Scheme 1b shows a step‐wise fabrication process of the BSA/anti‐CYFRA‐21‐1/APTES/ZrO_2_/ITO immunosensor along with the biochemical reaction.

## Supporting information

As a service to our authors and readers, this journal provides supporting information supplied by the authors. Such materials are peer reviewed and may be re‐organized for online delivery, but are not copy‐edited or typeset. Technical support issues arising from supporting information (other than missing files) should be addressed to the authors.

Supplementary
