# Supplementary material for: Biofunctionalized Nanostructured Zirconia for Biomedical Application: A Smart Approach for Oral Cancer Detection
Source: Adv Sci (Weinh). 2015 Jun 3;2(8):1500048. doi: 10.1002/advs.201500048 (PMC5115417; doi:10.1002/advs.201500048)
Supplement: Supplementary file 1 — Supplementary [file ADVS-2-0i-s001.pdf]

Copyright WILEY-VCH Verlag GmbH & Co. KGaA, 69469 Weinheim, Germany, 2015.

ADVANCED SCIENCE
Open Access

Supporting Information

for *Adv. Sci.*, DOI: 10.1002/advs.201500048

Biofunctionalized Nanostructured Zirconia for Biomedical Application: A Smart Approach for Oral Cancer Detection

*Suveen Kumar, Saurabh Kumar, Sachchidanand Tiwari, Saurabh Srivastava, Manish Srivastava, Birendra Kumar Yadav, Saroj Kumar, Thien Toan Tran, Ajay Kumar Dewan, Ashok Mulchandani, Jai Gopal Sharma, Sagar Maji, and Bansi Dhar Malhotra**

<u>**Supporting Information**</u>

Biofunctionalized Nanostructured Zirconia for Biomedical Application: A smart approach for Oral Cancer Detection

*Suveen Kumar, Saurabh Kumar, Sachchidanand Tiwari, Saurabh Srivastava, Manish Srivastava, Birendra Kumar Yadav, Saroj Kumar, Thien Toan Tran, Ajay Kumar Dewan, Ashok Mulchandani, Jai Gopal Sharma, Sagar Maji and Bansi Dhar Malhotra**

Nanobioelectronics Laboratory, Department of Biotechnology, Delhi Technological University, Delhi-110042, India
Email: bansi.malhotra@gmail.com

Manish Srivastava
Department of Physics and Astrophysics, University of Delhi- Delhi-110007, India

Birendra Kumar Yadav, Ajay Kumar Dewan
Rajiv Gandhi Cancer Institute and Research Centre, Rohini, Delhi-110085, India

Thien Toan Tran, Ashok Mulchandani
Department of Chemical and Environmental Engineering, University of California, Riverside, CA-92521, USA

Figure S1. XRD pattern of ZrO_2 nanoparticles before and after calcinations.

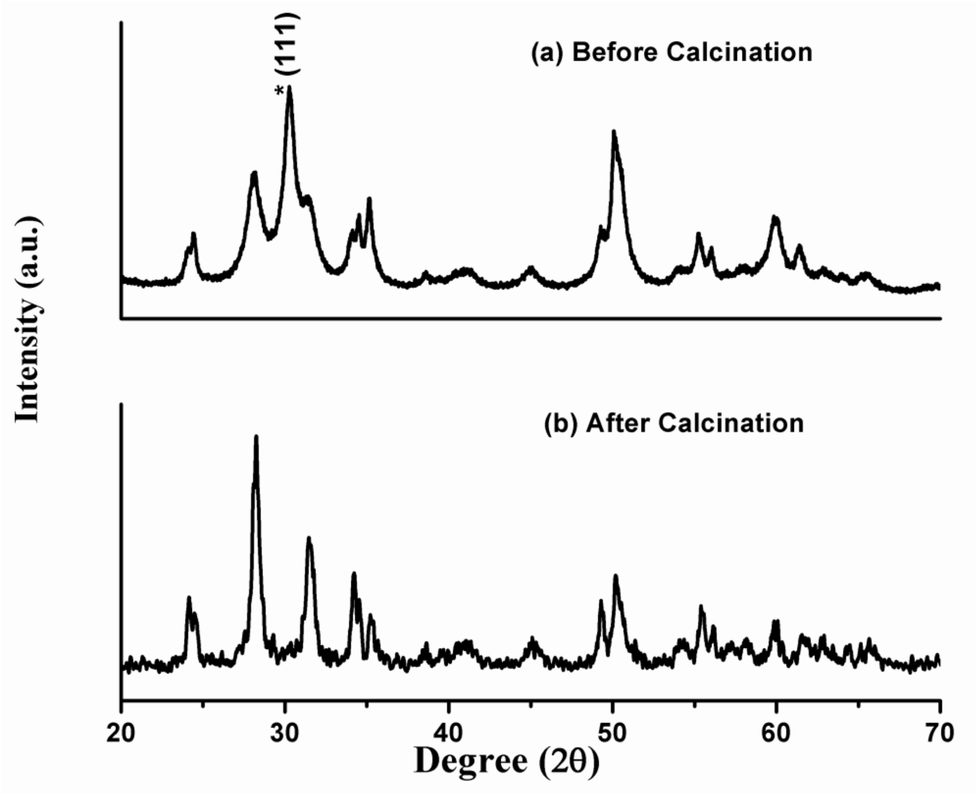

Figure S2. SEM micrograph shows side view of (a) APTES/ZrO_2/ITO and (b) anti-CYFRA-21-1/APTES/ZrO_2/ITO electrodes

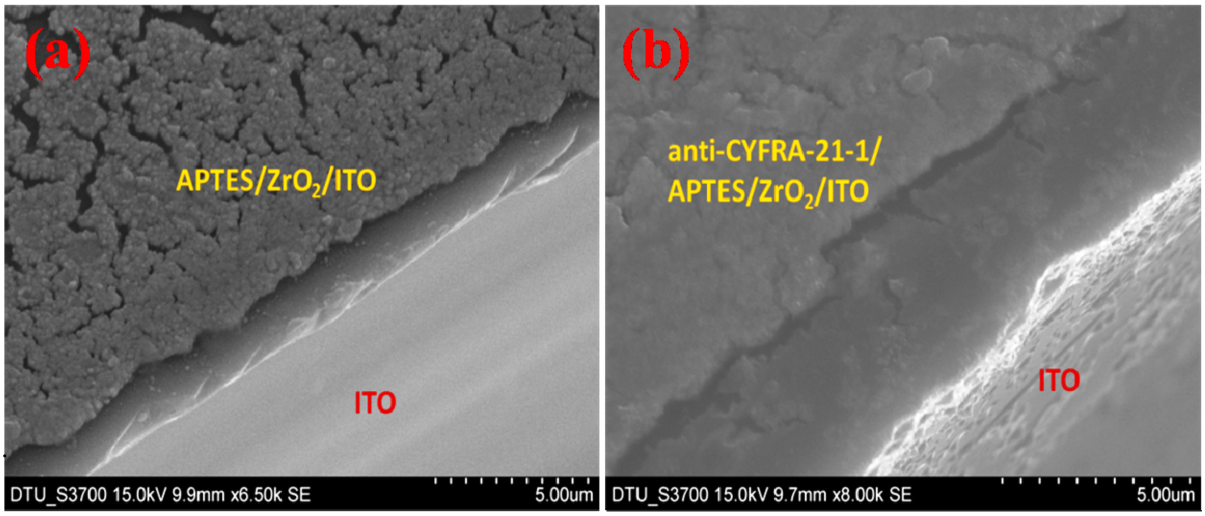

Figure S3. Current response of the BSA/anti-CYFRA-21-1/APTES/ZrO_2/ITO immunoelectrode as a function of pH.

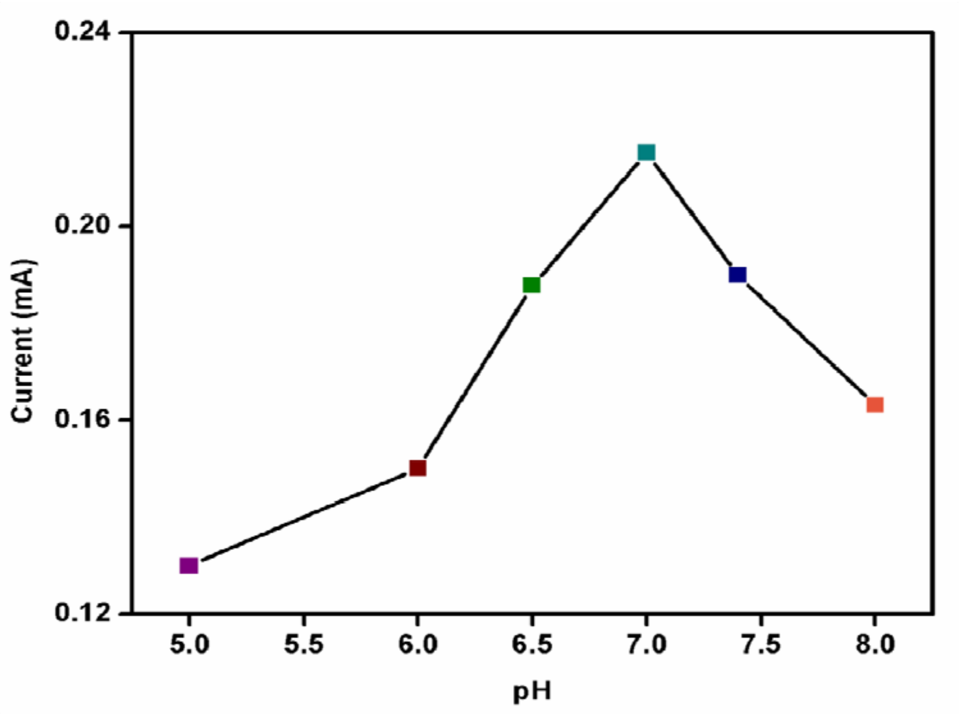

Figure S4. Cyclic voltammetry (CV) of APTES/ZrO_2/ITO electrode as a function of scan rate (10-100 mV/s). Magnitude of oxidation and reduction current response as a function of square root of scan rate (mV/s) (inset a), and difference of cathodic and anodic peak potential (ΔEp) as a function of square root of scan rate (inset b).

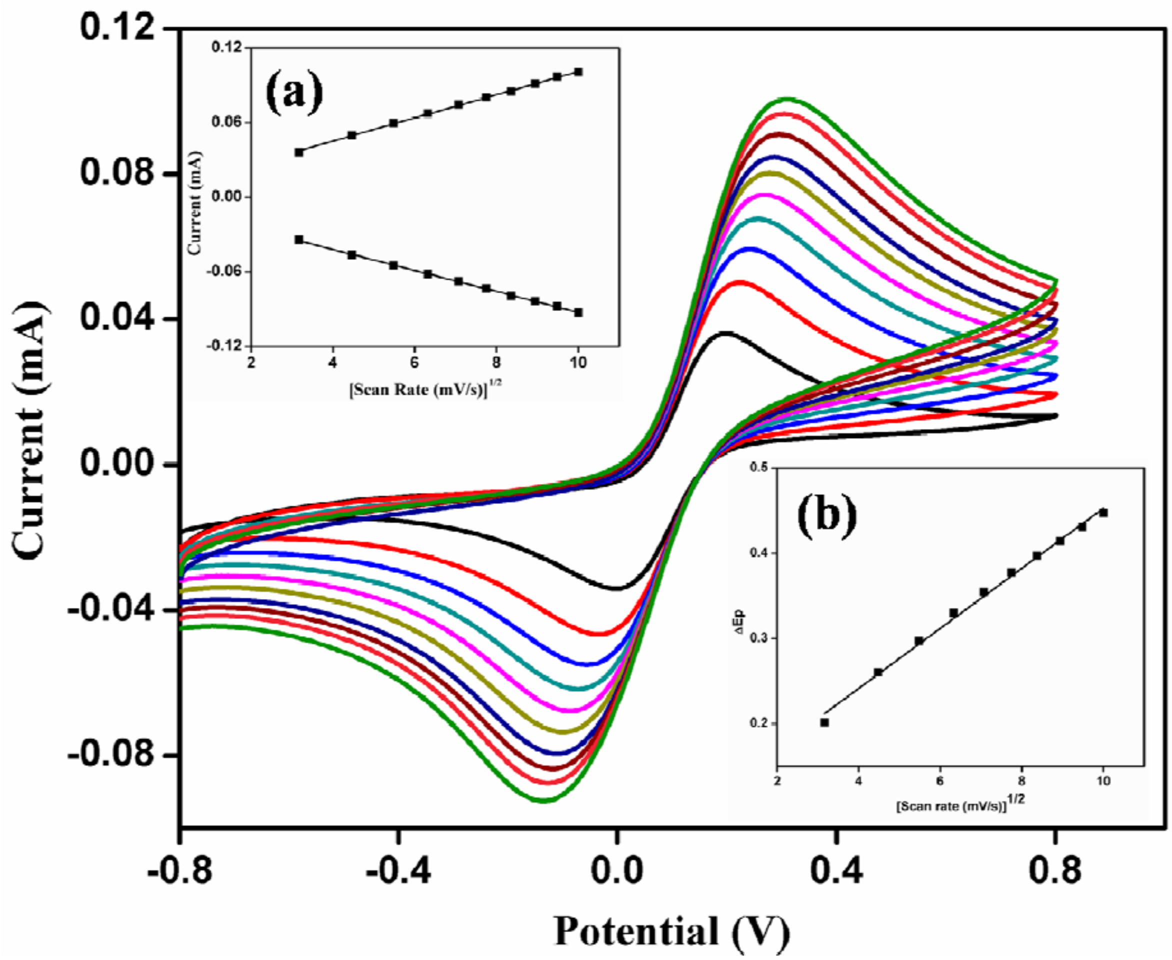

Figure S5. Cyclic voltammetry (CV) of BSA/anti-CYFRA-21-1/APTES/ZrO_2/ITO immunoelectrode as a function of scan rate (10-100 mV/s). Magnitude of oxidation and reduction current response as a function of square root of scan rate (mV/s) (inset a), and difference of cathodic and anodic peak potential (ΔEp) as a function of square root of scan rate (inset b).

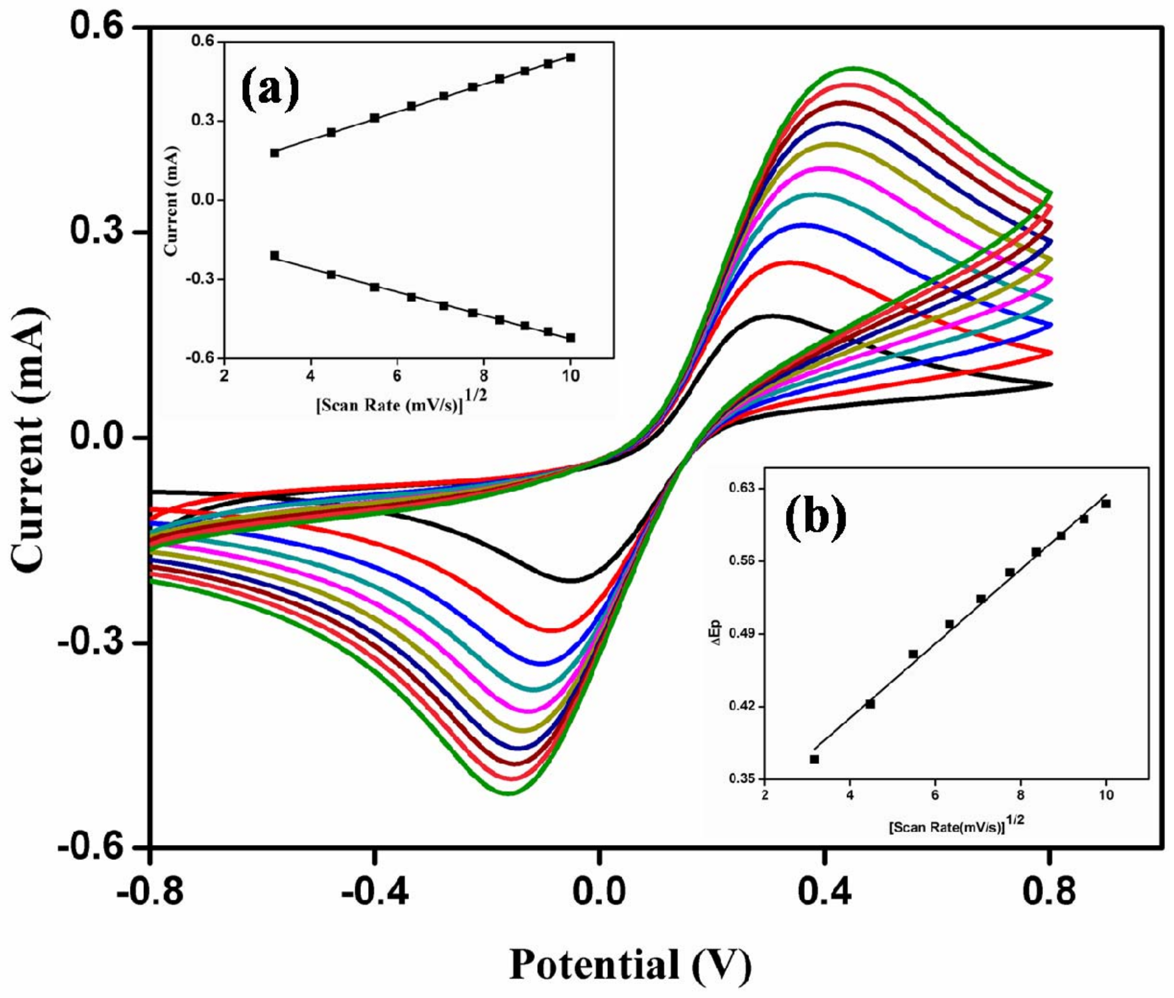

Figure S6. Incubation time studies for binding of CYFRA-21-1 with BSA/anti-CYFRA-21-1/APTES/ZrO_2/ITO immunoelectrode.

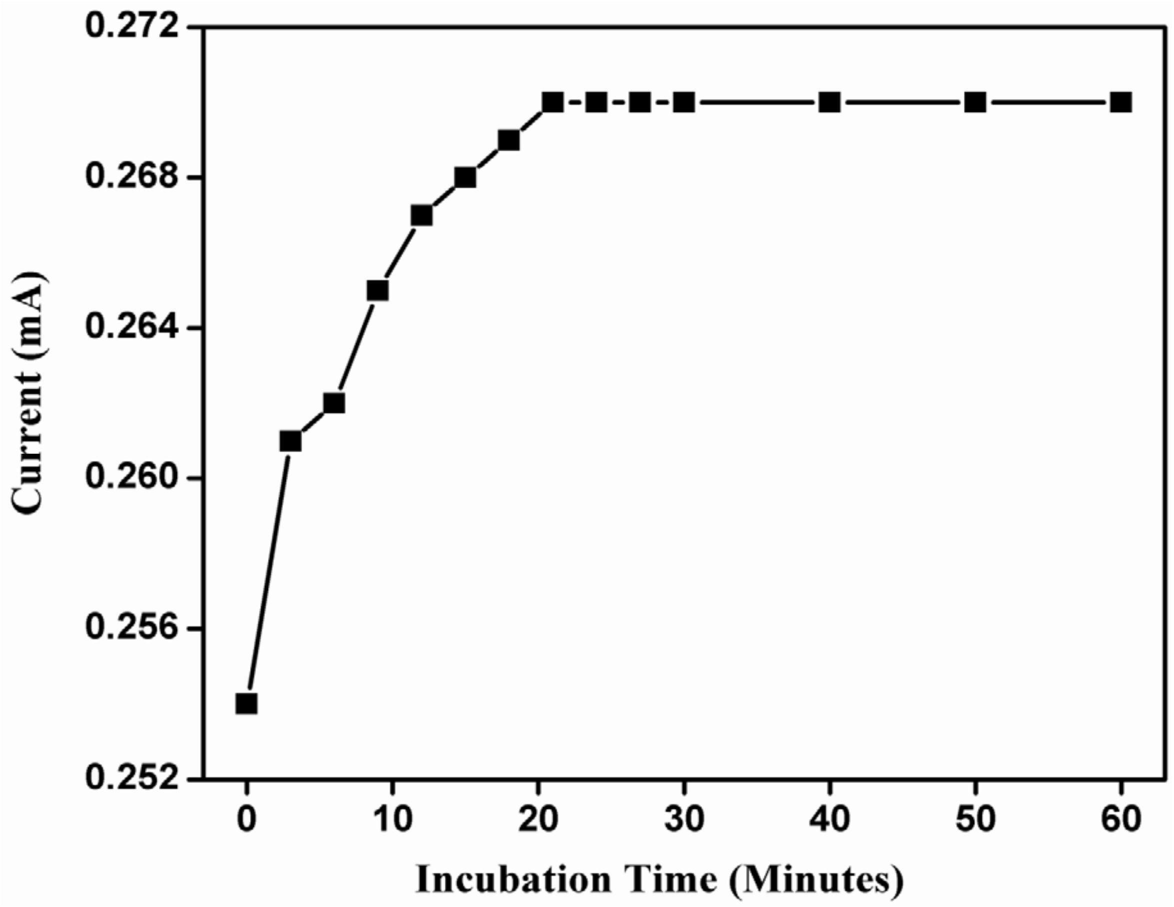

Figure S7. Control experiment (through electrochemical response study) of APTES/ZrO_2/ITO electrode as a function of CYFRA-21-1 concentration (0-16 ng mL^{-1}).

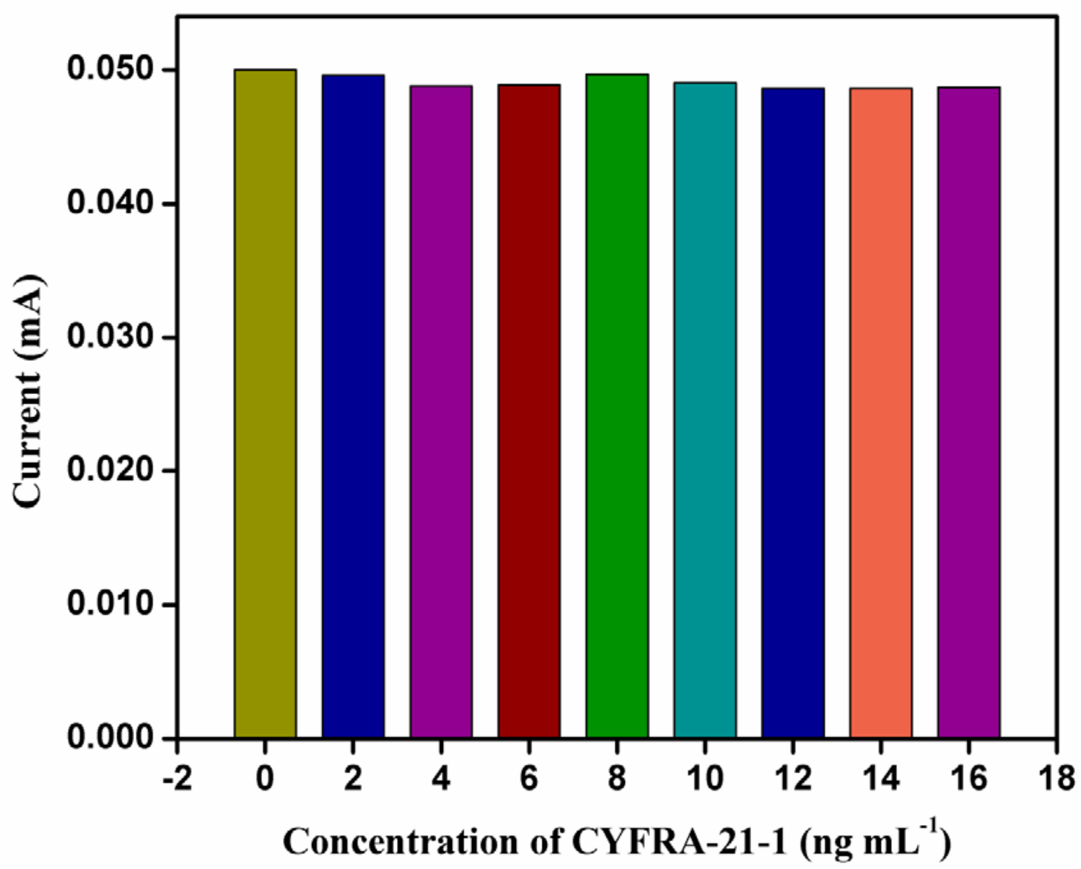

Figure S8. Interferent studies of BSA/anti-CYFRA-21-1/APTES/ZrO_2/ITO immunoelectrode.

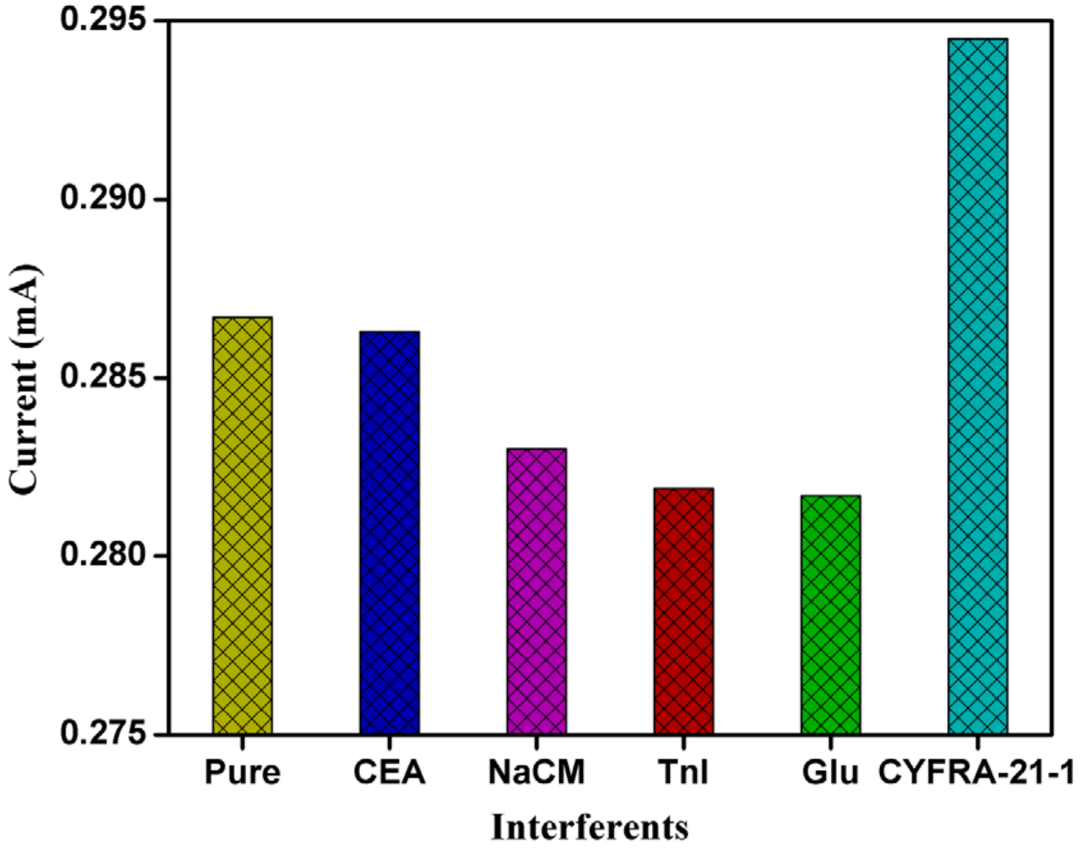

Figure S9. Shelf life studies of BSA/anti-CYFRA-21-1/APTES/ZrO_2/ITO immunoelectrode.

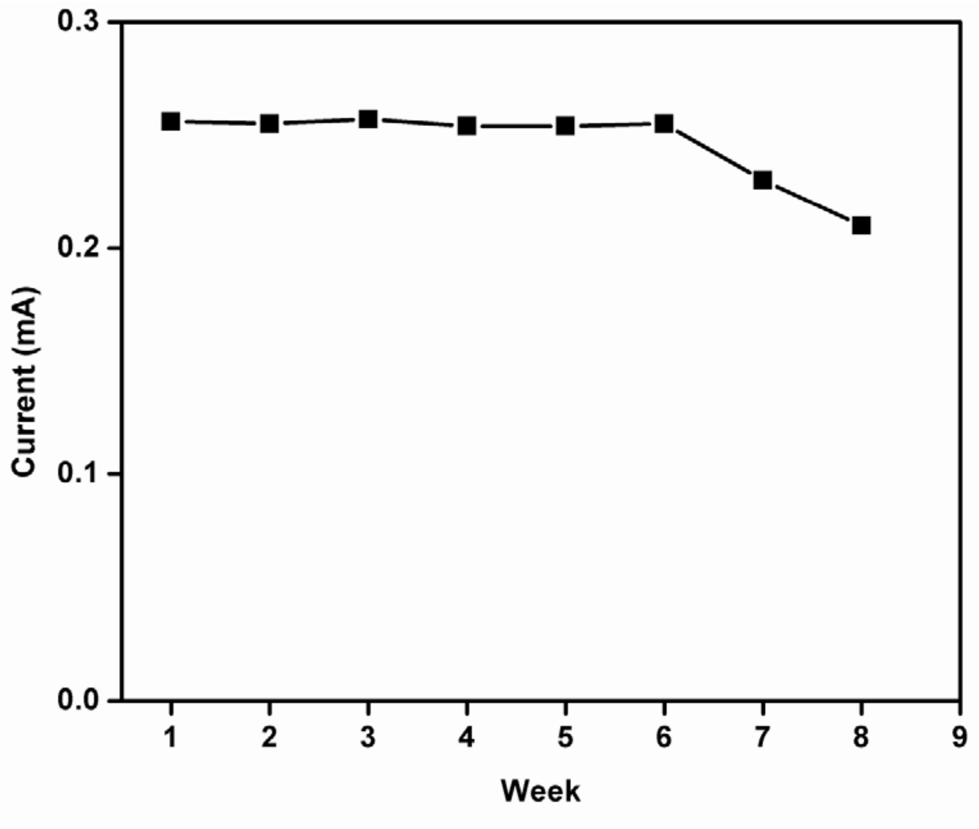

Figure S10. Effects of ions present in artificial saliva on electrochemical response study of BSA/anti-CYFRA-21-1/APTES/ZrO_2/ITO immunoelectrode.

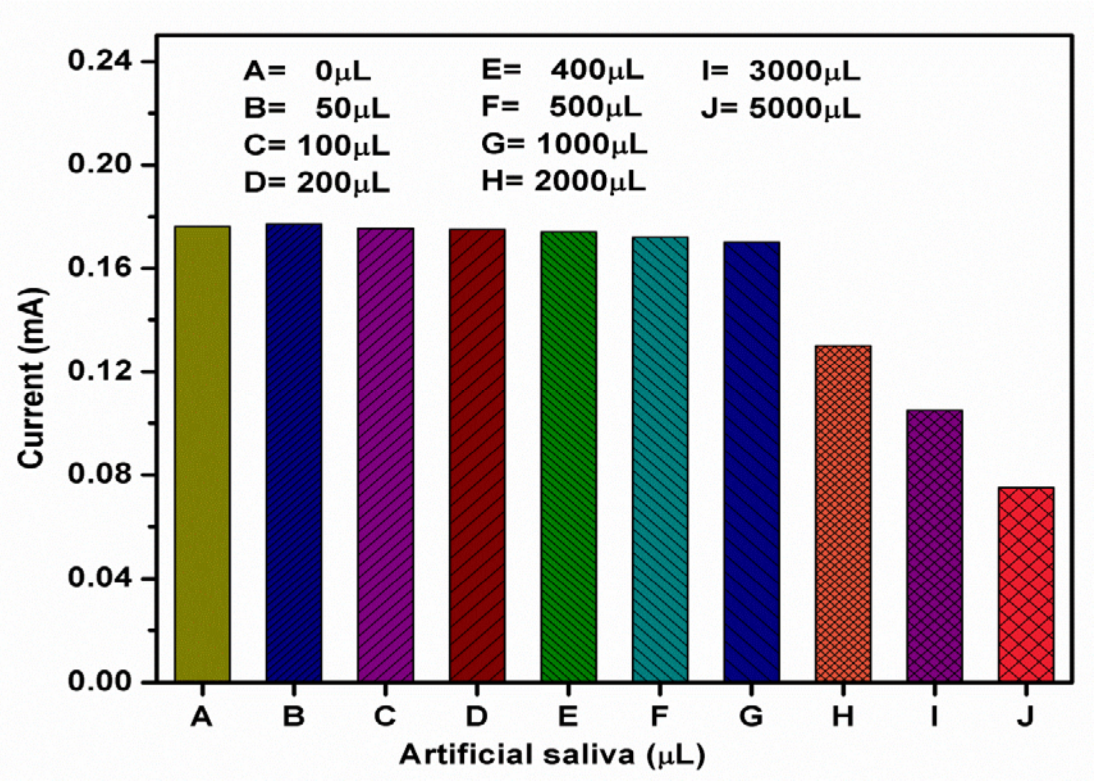

Figure S11. Comparative analysis of current response between standard sample and patient samples using fabricated BSA/anti-CYFRA-21-1/APTES/ZrO_2/ITO immunoelectrode.

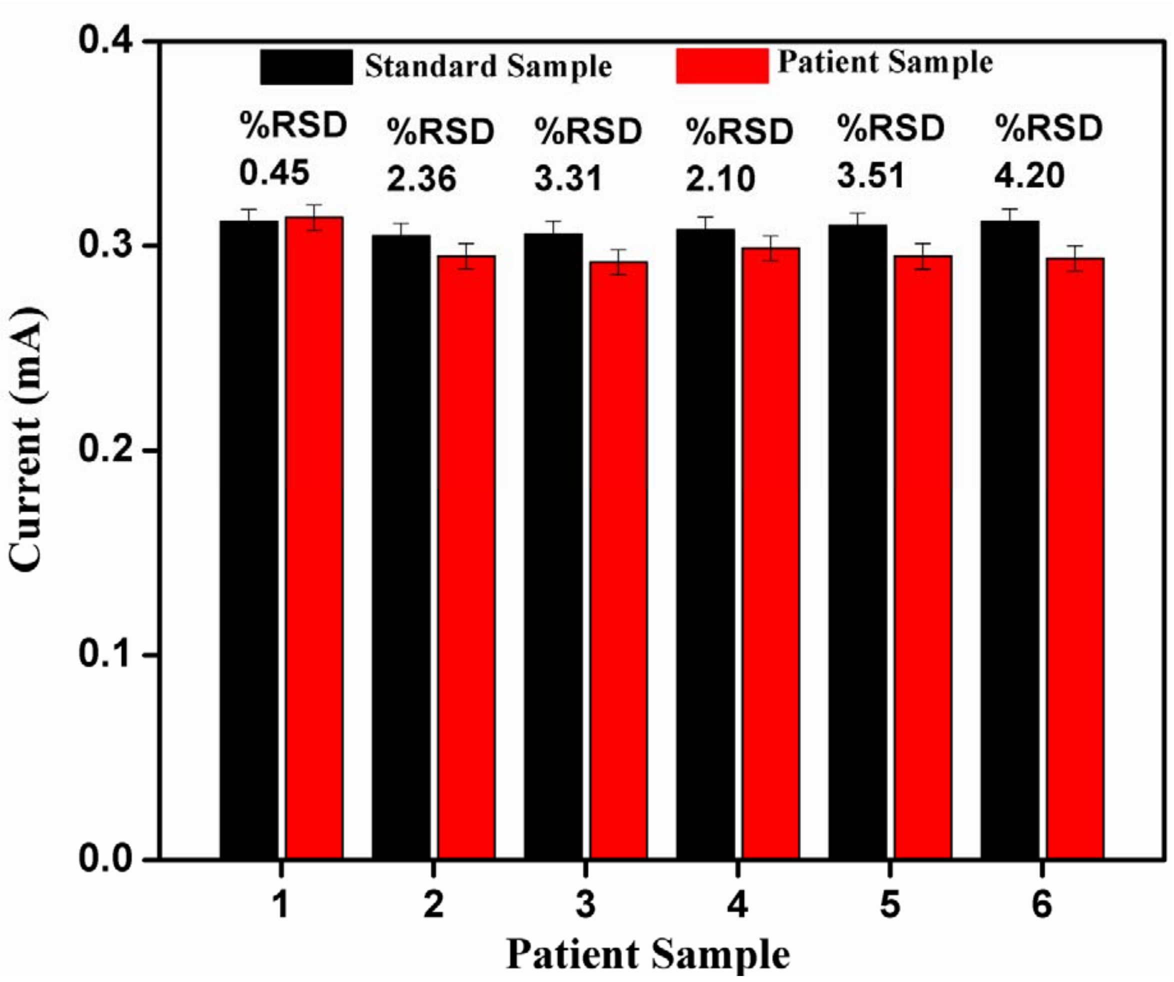

Table S1. Pathological data of oral cancer Patients

S. No.	Age	Sex	Habits (Tobacco chewing/ Smoking/alcohol)	Histological differentiation (Well differentiated (WD)/ Moderately differentiated (MD)/ Poorly differentiated)	Site of Lesion	Histological Type
1.	44	F	No Smoking, No Tobacco	Poorly Differentiated	Right Lobe of Thyroid	CA Papilliary Thyroid Carcinoma
2.	50	M	Alcohol only	Moderately Differentiated	Right side of Tongue (ulceroindurated)	Squamous Cell Carcinoma
3.	69	M	Smoking & alcohol	Moderately differentiated	Epiglottis	Squamous Cell Carcinoma
4.	61	M	Unknown	Moderately Differentiated	Right Side of Hard Palate	Squamous Cell Carcinoma
5.	49	M	Tobacco & Alcohol	Well differentiated	Tongue	Squamous Cell Carcinoma
6.	58	M	Tobacco & Alcohol	Moderately Differentiated	Left Tonsillar Fossa	Squamous Cell Carcinoma
